# Idiopathic hypertrophic pachymeningitis with anticardiolipin antibody: A case report

**DOI:** 10.1097/MD.0000000000024387

**Published:** 2021-01-15

**Authors:** Chi-Shun Wu, Hung-Ping Wang, Sheng-Feng Sung

**Affiliations:** a Division of Neurology; b Division of Rheumatology, Department of Internal Medicine, Ditmanson Medical Foundation Chia-Yi Christian Hospital, Taiwan.

**Keywords:** case report, erythrocyte sedimentation rate, headache, idiopathic hypertrophic pachymeningitis, immunosuppressant

## Abstract

**Rationale:**

Idiopathic hypertrophic pachymeningitis (IHP) is a rare neurological disorder without a definite etiology. Diagnosis is mainly based on exclusion of other etiologies.

**Patient concerns:**

A 41-year-old male patient presented with insidious onset headache of 3-month duration.

**Diagnoses:**

Contrast-enhanced brain magnetic resonance imaging (MRI) revealed diffuse pachymeningeal enhancement over bilateral cerebral hemispheres and the tentorium cerebelli. Lumbar puncture showed increased pressure, lymphocytic pleocytosis, and elevated protein level with normal glucose concentration. Blood tests detected elevated erythrocyte sedimentation rate (ESR) and C-reactive protein. Pathological examination of the dura mater from the right frontal convexity disclosed coarse collagenous deposition with focal lymphoid aggregation. After malignancy and infectious etiologies were excluded, a diagnosis of IHP was made.

**Interventions:**

Oral prednisolone and azathioprine followed by methotrexate were administered.

**Outcomes:**

During the 7-year follow-up period, although the patient was not totally headache-free, medical therapy significantly reduced the severity of headache. Follow-up MRI studies showed a reduction in meningeal enhancement and serial ESR measurements revealed a trend of improvement.

**Lessons:**

Methotrexate therapy may be considered in cases of steroid-resistant IHP. In addition to clinical evaluation, serial ESR testing may be considered to guide the treatment strategy and assess the response to therapy.

## 1 Introduction

Hypertrophic pachymeningitis is a rare neurological disorder characterized by localized or diffuse thickening of the dura mater. The disorder can be secondary to infections such as tuberculosis and syphilis, autoimmune disorders such as rheumatoid arthritis and vasculitis, sarcoidosis and neoplasms.^[1]^ When no identifiable etiology can be found, it is termed idiopathic hypertrophic pachymeningitis (IHP).^[1,2]^ According to a nationwide survey in Japan, its prevalence is less than 1/100,000.^[3]^ IHP can cause a variety of neurologic symptoms and signs including headache, loss of vision, and cranial nerve palsy.^[1,2]^ Among them, headache is the most common symptom,^[2,4,5]^ followed by ophthalmological manifestations, such as visual loss and diplopia.^[6,7]^ The typical brain magnetic resonance imaging (MRI) findings are characterized by prominent meningeal enhancement, which is also commonly seen in intracranial hypotension, carcinomatous meningeal involvement, tuberculosis meningitis, and autoimmune vasculitis disorders.^[8–10]^ Therefore, IHP is mainly a diagnosis of exclusion.

Because of the rarity of this disease, controlled studies of treatment of IHP seem to be impossible. Currently, there are no recommended treatment guidelines for IHP. In previously reported cases of IHP, the majority of them were treated with oral steroid with or without immunosuppressive agents, such as azathioprine and methotrexate.^[2,7]^ Besides, patient response to treatment was generally assessed by clinical evaluation, follow-up brain MRI, and serum inflammatory markers such as erythrocyte sedimentation rate (ESR) and C-reactive protein (CRP).^[1,2,7]^ Cases with long-term follow-up have been rarely reported in the literature. We herein presented a biopsy-proven case of IHP with 7 years of follow-up. A written informed consent was obtained from the patient for publication of this case report and accompanying images.

## 2 Case report

A 41-year-old man presented to the neurology clinic with insidious onset headache of 3-month duration. He had a history of hypertension. The headache was dull aching in bilateral frontal regions and was aggravated by lying supine and was worse at night. He also had episodic nausea with vomiting and progressive unsteady gait. He had never experienced fever during this illness. Non-contrast brain computed tomography performed at an outside hospital was reportedly normal. However, his headache severity had gradually worsened since symptoms onset.

**Figure 1:**
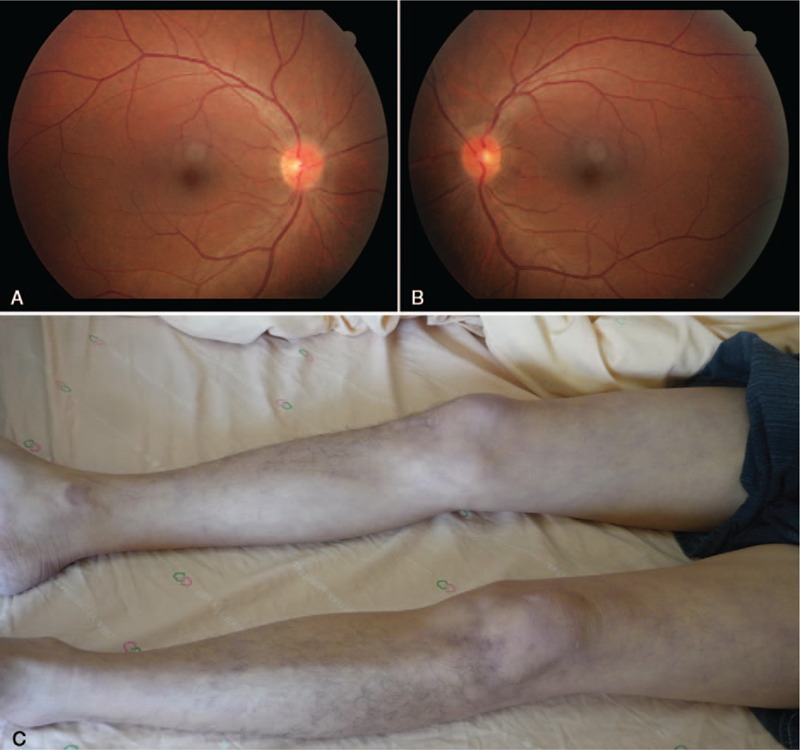
Bilateral papilledema on fundoscopy, right eye (A) and left eye (B). Livedo reticularis on bilateral legs (C).

Physical examinations revealed bilateral papilledema (Fig. 1A and B), a supple neck, gait ataxia, and lived reticular is on his legs (Fig. 1C). The laboratory workup revealed a normal hemogram and biochemistry, but an elevated ESR (91 mm/H) and CRP (25.8 mg/L). The rapid plasma reagin test for syphilis and serum cryptococcal antigen test were both negative. Hepatitis B virus surface antigen, anti-hepatitis C virus antibody, anti-human T lymphotropic virus type I/II antibodies, and anti-human immunodeficiency virus antibody were all negative. Serum complement levels and immunoglobulin (Ig) G4 were within normal limits. The autoimmune profile showed a negative rheumatoid factor, antinuclear antibody, cytoplasmic anti-neutrophil cytoplasmic antibody, anti-double-stranded DNA antibody, and anti-Ro/La antibodies. Antiphospholipid workup revealed negative anti-beta 2-glycoprotein I antibody and anticardiolipin IgG but positive anticardiolipin IgM (38.2 MPL; reference range, <12.5 MPL), which was persistently positive (26.2 MPL) on follow-up study.

**Figure 2:**
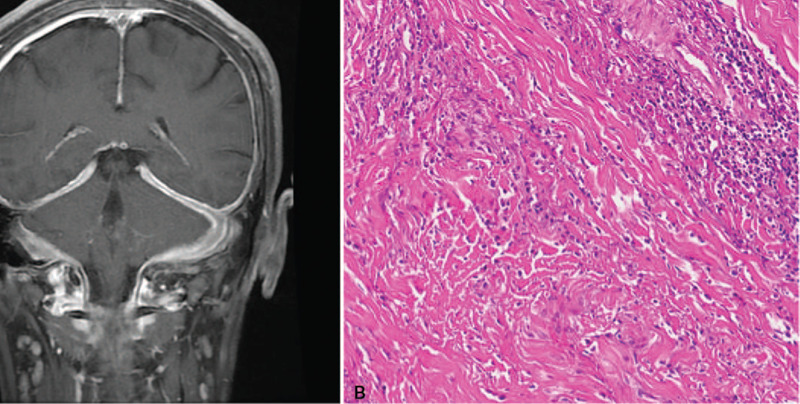
Diffuse dural enhancement over bilateral cerebral hemispheres and the tentorium cerebelli on contrast-enhanced T1-weighted brain magnetic resonance imaging (A). Biopsy specimen from the dura mater of the right frontal convexity showing coarse collagenous deposition and focal lymphoid aggregation (hematoxylin and eosin stain, 200X) (B).

Brain MRI disclosed diffuse thickening of pachymeninges with prominent contrast enhancement (Fig. 2A). Lumbar puncture showed an increased cerebrospinal fluid (CSF) pressure (230 mm H_2_O), lymphocytic pleocytosis (14 per mm^3^) and mildly elevated protein level (53.7 mg/dl; reference range 15–45 mg/dl) but normal glucose concentration. The results of CSF Gram stain, acid-fast stain, and India ink preparation were all negative.

In order to rule out carcinomatous meningeal involvement or chronic central nervous system infection, biopsy of the thickened dura mater from the right frontal convexity was performed. In the histological examination (hematoxylin and eosin stain, 200X), the meninges revealed fibrosis and thick, coarse collagen deposition with a moderate degree of lymphoid infiltrates (Fig. 2B). No granulomatous inflammation or vasculitis was found. No evidence of malignancy was observed. The acid-fast, periodic acid–Schiff, and Grocott methenamine silver stains failed to demonstrate any specific microorganisms. The microscopic characteristics could be consistent with pacchymeningitis. After malignancy and infectious etiologies were excluded, he was diagnosed with IHP according to the clinical presentation, brain MRI, and pathological findings.

**Figure 3:**
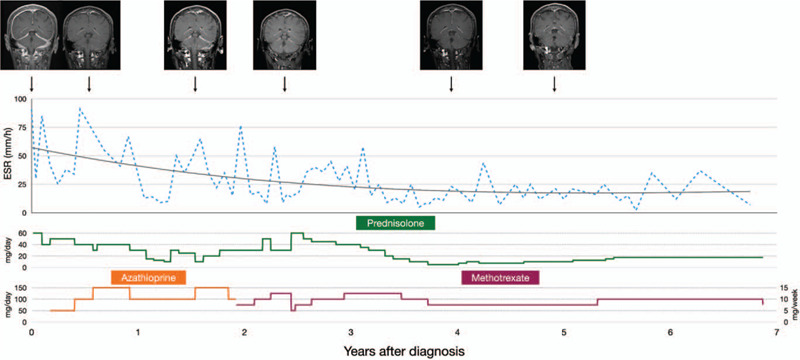
Serial contrast-enhanced T1-weighted brain magnetic resonance imaging and ESR levels in the treatment course of the patient.

He was started on treatment with oral prednisolone 60 mg daily, which significantly relieved his headaches and gait ataxia. However, Cushingoid appearance developed soon after starting steroid therapy. Tapering of prednisolone dose was thereby attempted. Even though his gait ataxia and lived reticular is disappeared completely, the intensity of his headaches fluctuated during the steroid taper. Consequently, oral azathioprine was added to the steroid therapy (Fig. 3). The doses of prednisolone (ranging from 10 mg per day to 60 mg per day) and azathioprine (ranging from 50 mg per day to 150 mg per day) were adjusted according to the severity of the headaches and ESR levels during the first 2 years of treatment. Because he was never headache-free during this period, azathioprine was switched to oral methotrexate (ranging from 5 mg per week to 12.5 mg per week) at approximately 2 years after diagnosis. The severity of his headaches still fluctuated and usually increased when the steroid dose was decreased. By increasing the dose of methotrexate, the dose of prednisolone was later reduced to below 20 mg daily at 3.5 years after starting treatment, thus minimizing the side effects of steroid therapy. During the 7-year follow-up period, serial brain MRI studies showed a reduction in meningeal enhancement and ESR measurements revealed a trend of improvement (Fig. 3).

## 3 Discussion

IHP is rarely encountered in clinical practice. Only sporadic cases or small case series have been reported.^[1,2,4–7,11]^ Although the treatment strategy and outcome vary widely among patients,^[1,2,7]^ the majority of cases of IHP were treated with steroids or a combination of steroids and immunosuppressive agents.^[2,5–7,11]^ The clinical response to treatment was often evaluated by clinical presentation and follow-up brain MRI.^[1,2]^

Our patient has been followed up for 7 years with serial contrast-enhanced brain MRI studies and ESR testing. Although a reduction in meningeal enhancement was observed on follow-up MRI images, the interpretation of MRI findings is somewhat subjective. A substantial proportion of patients with IHP have elevated ESR levels prior to steroid or immunosuppressant therapy.^[1,2,7]^ Good clinical response to treatment generally correlates with a reduction in ESR levels,^[1,7]^ as seen in our patient. As compared to contrast-enhanced brain MRI, ESR testing is much more available and affordable. This might suggest that ESR levels could be considered an indicator for treatment response in IHP patients with elevated ESR. Although clinical symptoms are essential in the evaluation of treatment response, ESR testing might help clinicians guide the adjustment of treatment regimens especially when MRI is not available or affordable.

Interestingly, our patient was found positive for anticardiolipin IgM. A literature search revealed sporadic cases of hypertrophic pachymeningitis associated with antiphospholipid syndrome.^[12,13]^ In addition, a case of IgG4-related pachymeningitis with underlying antiphospholipid syndrome has been reported.^[14]^ However, our patient had never experienced thrombotic events suggestive of antiphospholipid syndrome, including deep vein thrombosis and stroke even though he did have lived reticular is. Furthermore, pathological examination of the dura mater of our patient did not show the typical findings of dense lymphoplasmacytic infiltrates seen in cases of IgG4-related pachymeningitis.^[15,16]^ Therefore, we believe a diagnosis of IHP is appropriate for our patient. More clinical evidence is required to establish whether antiphospholipid antibodies-associated hypertrophic pachymeningitis is a distinct disease entity.

Uchida et al reported a case of steroid-resistant IHP, who had good clinical response to weekly low-dose methotrexate therapy.^[11]^ In our patient, the dose of oral prednisolone could not be tapered even though oral azathioprine was added. Nevertheless, after azathioprine was switched to methotrexate, the dose of oral prednisolone was successfully reduced to below 20 mg daily. Successful treatment with methotrexate was also reported in a case of steroid-resistant IgG4-related pachymeningitis.^[17]^ Weekly low-dose oral methotrexate therapy seemed to improve the clinical course of our patient.

In conclusion, we presented a case of biopsy-proven IHP with long-term follow-up. The patient improved in clinical symptoms after being treated with steroid therapy but experienced side effects and worsening of headache during dose tapering. After weekly low-dose oral methotrexate was added, the dose of oral prednisolone was successfully reduced. A reduction in ESR levels was observed in association with clinical response to treatment. Methotrexate therapy may be considered in cases of steroid-resistant IHP. In addition to clinical evaluation, serial ESR testing may be considered to guide the treatment strategy and assess the response to therapy.

## Acknowledgments

The authors would like to thank Ms Li-Ying Sung for English language editing.

## Author contributions

**Conceptualization:** Chi-Shun Wu, Hung-Ping Wang, Sheng-Feng Sung.

**Investigation:** Chi-Shun Wu, Hung-Ping Wang, Sheng-Feng Sung.

**Visualization:** Sheng-Feng Sung.

**Writing – original draft:** Chi-Shun Wu, Hung-Ping Wang.

**Writing – review & editing:** Sheng-Feng Sung.

## Abbreviations

Abbreviations: CRP = C-reactive protein, CSF = cerebrospinal fluid, ESR = erythrocyte sedimentation rate, IHP = Idiopathic hypertrophic pachymeningitis, MRI = magnetic resonance imaging.
